# Elevated antibiotic resistance of Sudanese urinary tract infection bacteria

**DOI:** 10.17179/excli2017-424

**Published:** 2017-08-07

**Authors:** Amir Saeed, Shadia A. Hamid, Magdi Bayoumi, Salah Shanan, Sultan Alouffi, Samir A. Alharbi, Fawaz D. Alshammari, Hadi Abd

**Affiliations:** 1 University of Hail, College of Applied Medical Sciences, Department of Clinical Laboratory Science, Hail, Kingdom of Saudi Arabia; 2 University of Medical Sciences and Technology, Faculty of Medical Laboratory Sciences, Department of Microbiology, Khartoum 11111, Sudan; 3 Karolinska Institute, Department of Laboratory Medicine, Division of Medical Microbiology, Stockholm, Sweden; 4 Al-Quweayiyah, Shaqra University, College of Applied Medical Sciences, Department of Medical Laboratory Sciences, Kingdom of Saudi Arabia

**Keywords:** urinary tract infections, bacteria, antibiotic resistance

## Abstract

This study determined the prevalence of urinary tract infections in the Sudanese state of Khartoum and antimicrobial susceptibility pattern of isolated bacterial species. 200 adult patient urine specimens were collected and cultivated to identify the growing bacteria and their susceptibility to antibiotics. 35 % of specimens had significant bacterial growth. The most frequent isolates in this study were *E*. *coli*, *E. faecalis *and *S. aureus. *Most of the isolates were resistant to many antibiotics; Gram-negative and Gram-positive isolates were resistant to 67 % and 44 % of the examined antibiotics, respectively. *E. coli *was the most frequent bacterium in the studied samples and it was highly resistant to first-line antibiotics. The most resistant bacteria isolated were *Pseudomonas *species and the lowest was for *S. saprophyticus. *The results highlighted the need for knowledge about antibiotic susceptibility profile of the bacteria causing UTI prior to antibiotic prescription in order to ensure optimal treatment.

## Introduction

Urinary tract infection (UTI) is the second most common infectious presenting in community practice. Worldwide, about 150 million people are diagnosed with UTI each year (Gonzalez and Schaeffer, 1999[10]). Almost 95 % of cases of UTIs are caused by bacteria (Bishop et al., 2007[4]). Several studies show geographic variations in etiologic agents of UTIs and their resistance patterns to antibiotics (Gupta, 2003[11]; Akoachere et al., 2012[2]). A study from 2006 carried out by Theodore (2006[24]) in Nigeria found 141 out of 181 (77.9 %) urine samples gave significant growth and the common isolates were *E. coli, K. pneumoniae *and *S. aureus *(Theodore, 2006[24]; Ebie et al., 2001[8]; Burbige and Retik, 1984[6])*. *De Francesco et al. (2007[7]) found that the most common causative agents of UTIs in Italy were *E. coli*, *E. faecalis*, *K. pneumoniae *and *P. mirabilis*. In this context, Ahmed et al. (2000[1]) reported that the most common urinary bacteria isolated in Sudan were *E. coli*, *Klebsiella pneumoniae*, and *Proteus mirabilis *(Ahmed et al., 2000[1]).

UTIs are often treated with broad-spectrum antibiotics that affect both Gram-positive and Gram-negative bacteria. However it might be more appropriate to use an antibiotic with a narrow spectrum activity that affects only Gram-positive or Gram-negative bacteria because of concerns about infection with resistant organisms. Moreover, the extensive uses of antimicrobial agents have invariably resulted in the development of antibiotic resistance, which has become a major problem worldwide (Kumar et al., 2006[17]).

The etiology of UTI and their antibiotic resistance have been changed over the past years, both in community and hospital-contracted infection (Manges et al., 2006[19]; Kahan et al., 2006[16]). Gutpa et al. (1999[13]) noted an alarming frequency of bacterial resistance. Most routinely used antibiotics had an overall sensitivity of less than 25 %; these included penicillin, ampicillin, oxacillin, streptomycin, tetracycline, chloramphenicol, carbenicillin and sulphonamides (Gupta et al., 1999[13]). It was shown that resistance of *E. coli *and other uropathogens to ß-lactams, such as ampicillin, and the first-generation cephalosporins has continued to increase in the past decade and now approaches 40 % in most studies (Gupta et al., 1999[13]).

Despite most Gram-negative uropathogens are still susceptible to the combination of amoxicillin-clavulanate, the expense and gastrointestinal side effects of this drug make it a less desirable choice for empirical treatment of uncomplicated UTI (Gupta et al., 2001[12]). However, it has been suggested that the failure rate with this drug is high when the bacteria is resistant to ampicillin but susceptible to amoxicillin-clavulanate (Hooton and Stamm, 1997[15]). Whatever, fluoroquinolones are preferred, as initial agents for empiric therapy of UTI in area where resistance is likely to be of concern (Schaeffer, 2002[21]; Biswas et al., 2006[5]), since they have high bacteriological and clinical cure rates, as well as low rates of resistance, among most common uropathogens (Goldstein, 2000[9]; Gupta et al., 2002[14]; Tankhiwale et al., 2004[23]).

The previously reviewed studies uncovered geographic variations in etiologic agents of UTIs and their resistance patterns to antibiotics (Gonzalez and Schaeffer, 1999[10]; Bishop et al., 2007[4]; Gupta, 2003[11]; Akoachere et al., 2012[2]), however, in Sudan Ahmed et al. (2000[1]) found that the most common urinary isolates were highly resistant when they were tested against ampicillin, amoxicillin, co-trimoxazole, tetracycline, sulfonamide, trimethoprim, streptomycin, and carbenicillin (Gupta et al., 2001[12]). Unfortunately, there is not much information on etiology and resistance pattern of community acquired UTIs in Sudan is available, but helpfully, area-specific monitoring studies providing knowledge about the type of pathogens responsible for UTIs and their resistance patterns may help the clinician to choose the right empirical treatment (Beyene and Tsegaye, 2011[3]). Here we sought to determine prevalence of urinary tract infections in Khartoum state and antimicrobial susceptibility pattern of isolated bacterial species.

## Material and Methods

### Study design, area, and sample collection

The study was a cross-sectional hospital based study. It was conducted in the academy teaching hospital, Khartoum teaching hospital, The Academy Charity Hospital and Yastabshiroon medical centre. It was carried out on samples from 200 subjects of unknown sex and age taken between August and November 2008, at the University of Medical Sciences and Technology, Microbiology Laboratory. Two hundred mid-stream urine specimens were collected, as aseptically as possible, in a sterile wide-mouth container. It was not known whether the submitted urine came from patients with symptomatic upper or lower UTI or from patients with asymptomatic bacteriuria or whether the urinary infection was complicated or uncomplicated. Since urine itself is a good culture medium, all specimens were processed by the laboratory within 2 hours of collection, or kept refrigerated at 4 ºC until delivery to the laboratory, and subsequently processed no more than 18 hours after initial collection.

Whenever possible, urine specimens for culture were collected in the morning. The patient was advised the night before to refrain from urinating before specimen collection.

### Cultivation of urine samples, identification of growing bacteria and susceptibility to antibiotics

The urine samples were cultured on MacConkey and CLED agar plates and incubated aerobically at 37 °C overnight. Identification of growing bacteria was performed according to standard bacteriological methods. Sensitivity of bacteria to each antibiotic was carried out by measuring the diameter of inhibition zone of bacterial growth around the disc according to standards for antimicrobial disk susceptibility tests of National Committee for Clinical Laboratory Standards (1997[20]).

### Statistical analyses

A chi-2 test was used for comparison between numbers of susceptible and resistant isolated bacterial species in addition to compare antimicrobial resistance rate between Gram-negative and Gram-positive isolated bacteria. A p value of ≤ 0.05 was considered significant. 

## Results

### Prevalence and frequency of bacteria in urinary infection patient samples

Out of the 200 urine specimens, 69 had significant bacterial growth giving so overall, bacterial prevalence was 34.5 %. The contaminated samples contained *Escherichia coli*, *Enterococcus faecalis*, *Staphylococcus aureus*, *Proteus mirabilis*, *Klebsiella pneumoniae*, *Pseudomonas spp*. and *Staphylococcus saprophyticus *at 54, 19, 13, 4, 4, 3 and 3 % respectively (Table 1(Tab. 1)). All infections were monocultures; no cases of multiple infections were observed.

### Antimicrobial susceptibility of the isolated Gram-negative bacteria

To determine numbers and rates of susceptible and resistant bacteria, the antimicrobial susceptibility test was performed for the isolates of the four Gram-negative bacteria that exposed to 14 common relevant antibiotics: ampicillin, amikacin, cefotaxime, ciprofloxacin, ceftriaxone, amoxicillin, chloramphenicol, gentamicin, nalidixic acid, imipenem, norofloxacin, nitrofurantoin, co-trimoxazole and tetracycline. Numbers and rates of susceptible or resistant isolated Gram-negative bacteria were listed in Table 2(Tab. 2). *E. coli, K. pneumoniae, P. mirabilis *and* Pseudomonas* species showed high susceptible rates to gentamicin (Table 2(Tab. 2)). In contrast, these bacterial species were found to have high resistant rates against ampicillin, amikacin, amoxicillin, nitrofurantoin, co-trimoxazole and tetracycline. Unfortunately, *Pseudomonas* species isolates were resistant to all used antibiotics in this study except gentamicin (Table 2(Tab. 2)).

### Antimicrobial susceptibility of the isolated Gram-positive bacteria

To determine rates of susceptible and resistant bacteria, the antimicrobial susceptibility test was performed for the isolates of the three gram-positive bacteria that exposed to 14 common relevant antibiotics: ampicillin, vancomycin, cefotaxime, ciprofloxacin, amoxicillin, augmentin, chloramphenicol, erythromycin, nalidixic acid, imipenem, norofloxacin, nitrofurantoin, co-trimoxazole and penicillin. Numbers and rates of suscep-tible or resistant isolated Gram-positive bacteria were listed in Table 3(Tab. 3). *S. aureus*, *S. saprophyticus* and *E. faecalis* showed high susceptible rates to vancomycin, ciprofloxacin, amoxicillin and norofloxacin. Fortunately, *S. saprophyticus* was highly sensitive to all used antibiotics in this study (Table 3(Tab. 3)). However, *S. aureus* and *E. faecalis* isolates were resistant against cefotaxime, augmentin, erythromycin, nalidixic acid, co-trimoxazole and penicillin (Table 3(Tab. 3)).

### Comparison between numbers of susceptible and resistant isolates to 14 utilized antibiotics

Comparison between numbers of sensitive and resistant Gram-negative isolates to the utilised 14 antibiotics disclosed that *E. coli*, *K. pneumoniae, P. mirabilis *and *Pseudomonas *species were sensitive to 35 %, 26 %, 31 % and 21 % and resistant to 65 %, 74 %, 69 % and 79 % of the antibiotics, respectively. There was a very highly significant difference between the numbers of susceptible and resistant Gram-negative isolates (*p *˂ 0.0001). However, the total numbers of Gram-negative isolates were sensitive to 33 % and resistant to 67 % of the 14 antibiotics used in the study (Table 4(Tab. 4)).

Comparison between numbers of sensitive and resistant Gram-positive isolates to the relevant utilised 14 antibiotics disclosed that *E. faecalis*, *S. aureus *and *S. saprophyticus *were sensitive to 55.5 %, 48 % and 100 % and resistant to 44.5 %, 52 % and 0.0 % of the antibiotics, respectively. There was a highly significant difference between the numbers of susceptible and resistant Gram-positive isolates (*p *˂ 0.001). Whatever, the total number of Gram-positive isolates was sensitive to 56 % and resistant to 44 % of the 14 antibiotics used in the study (Table 4(Tab. 4)).

## Discussion

Here we determined the prevalence and frequency of antimicrobial susceptibility of bacterial species isolated from urine samples given by 200 adult Sudanese patients who presented with urinary disorders.

We found that 69 (34.5 %) samples had significant bacterial growth and the common isolates were *E. coli, E. faecalis *and *S. aureus*. Prevalence of our findings was less than the prevalence of a Nigerian study from 2006 carried out by Theodore who found 141 out of 181(77.9 %) urine samples gave significant growth and the common isolates were *E. coli, K. pneumoniae *and *S. aureus *(Theodore, 2006[24]; Ebie et al., 2001[8]; Burbige et al., 1984[6])*. *In this context, Ahmed et al. (2000[1]) from Sudan stated that the most common urinary bacteria isolated in his study were *E. coli*, *Klebsiella pneumoniae*, and *Proteus mirabilis.*

Our results are most similar to those of De Francesco et al. (2007[7]) in Italy, who found that the most common causative agents of UTIs were *E. coli*, *E. faecalis*, *K. pneumoniae *and *P. mirabilis*.

The common urinary bacteria isolated in our study were highly resistant to a number of the antimicrobial agents used, including ampicillin, amoxicillin, chloramphenicol, co-trimoxazole, naladixic acid, tetracycline and nitrofurantoin, in agreement with the study performed by Ahmed et al. (2000[1]) in Sudan, the most common urinary isolates were highly resistant when they were tested against ampicillin, amoxicillin, co-trimoxazole, tetracycline, sulfonamide, trimethoprim, streptomycin, and carbenicillin. He found also that *E. coli *had a relatively low rate of resistance to nitrofurantoin (17 %) (Ahmed et al., 2000[1]), but unfortunately our results uncovered that *E. coli *resistance to nitrofurantoin increased to 65 %. In this context, Gupta et al. (1999[13]) noted an alarming frequency of bacterial resistance. He found that most routinely used antibiotics had an overall sensitivity of less than 25 %; these included penicillin, ampicillin, oxacillin, streptomycin, tetracycline, chloramphenicol, carbenicillin and sulphonamides (Gupta et al., 1999[13]). Unfortunately, our results found that *E. coli*, *K. pneumoniae*, *P. mirabilis *and *Pseudomonas* species had an individual elevated resistance to the most of the utilized antibiotics since these resistance rates were 65 %, 74 %, 69 % and 79 %, respectively, giving an overall resistance rate of 67 % (Table 4(Tab. 4)).

Nitrofurantoin and the fluoroquinolones are still effective *in vitro* against most *E. coli *isolates that cause uncomplicated community-acquired UTI. Moreover, nitrofurantoin is less active against non *E. coli *Gram-negative rods and inactive against *Proteus *and *Pseudomonas *species (Gupta et al., 1999[13]; Sham et al., 2001[22]) and these findings were quite related to ours since 33 % of *P. mirabilis *was sensitive to nitrofurantoin and both *K. pneumoniae *and *Pseudomonas* species were resistant (Table 2(Tab. 2)) to give an overall sensitivity rate of 11 % which was less than 25 % reported by Gupta et al. (1999[13]).

In our study, the isolated Gram-positive bacteria were more sensitive to the antibiotics than Gram-negative isolates. It was found that *E. faecalis*, *S. aureus *and *S. saprophyticus *were sensitive to 55.5 %, 48 % and 100 % and resistant to 44.5 %, 52 % and 0.0 % of the relevant antibiotics. In comparison, The Gram-negative isolates *E. coli*, *K. pneumoniae, P. mirabilis *and Pseudomonas species were sensitive to 35 %, 26 %, 31 % and 21 % and resistant to 65 %, 74 %, 69 % and 79 % of the antibiotics (Table 4(Tab. 4)). However, the overall numbers of Gram-positive isolates were susceptible to 56 % and resistant to 44 % of the relevant 14 antibiotics, while the overall numbers of Gram-negative isolates were susceptible to 41 % and resistant to 59 % of the antibiotics (*p *˂ 0.0001).

Finally, the Gram-positive isolates were most susceptible to vancomycin, ciprofloxacin and augmentin while the Gram-negative isolates were most sensitive to ciprofloxacin, ceftriaxone and gentamicin (Tables 2(Tab. 2) and 3(Tab. 3)). The resistance observed among most organisms against the utilized antibiotics might be because these antibiotics have been in use for a long period and must have been abused and as a result the organisms must have developed mechanisms of circumventing their mode of action.

Here we show that bacteria in Sudanese urinary infections frequently develop resistance against 14 antimicrobial agents and thus we highlight a serious problem facing health authorities. However, we need more studies to uncover mechanisms behind this resistance. A better understanding of these *in situ *processes is required in order to control the development, transmission, and evolution of antibiotic resistant genes (Lin et al., 2015[18]). To develop new antibiotics, it is imperative to study the molecular basis of resistance development so that we can prevent and overcome antibiotic resistance by targeting resistance mechanisms. This will also make the existing and novel antibiotics more effective and sustainable (Lin et al., 2015[18]).

## Conclusions

*E. coli *was the most frequent bacterium in the studied urine samples and it was highly resistant to the most utilized antibiotics as were the other bacteria identified in this study. Our data highlight the need for developing local guidelines where elevated resistance to antibiotics should influence prescribing decisions.

## Conflict of interest

The authors declare that they have no conflict of interest.

## Figures and Tables

**Table 1:**
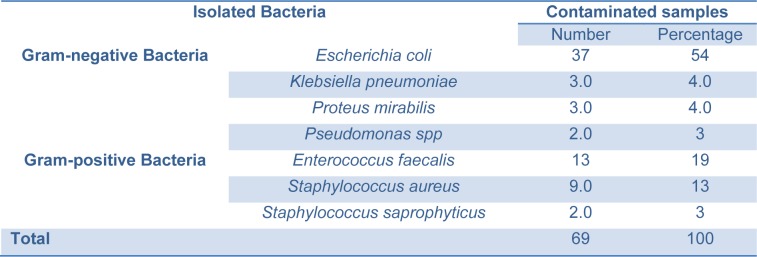
Number and frequency of isolated bacterial species from urine samples

**Table 2:**
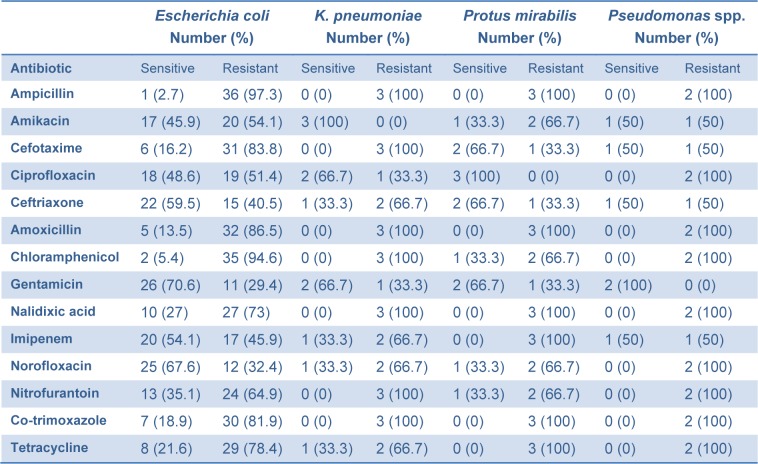
Antimicrobial susceptibility of Gram-negative isolates to tested antibiotics

**Table 3:**
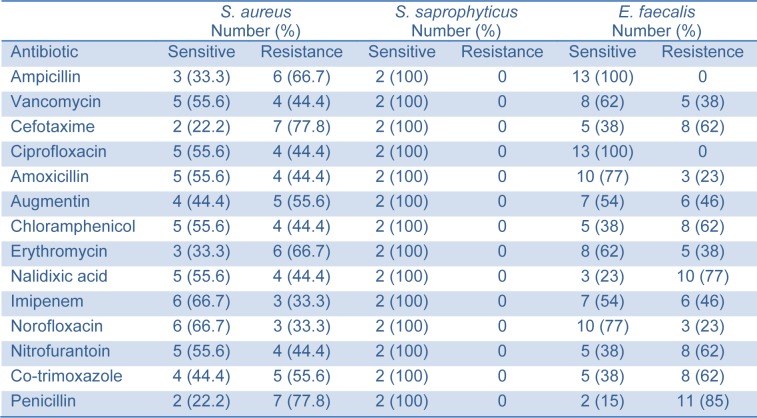
Antimicrobial susceptibility of Gram-positive isolates to tested antibiotics

**Table 4:**
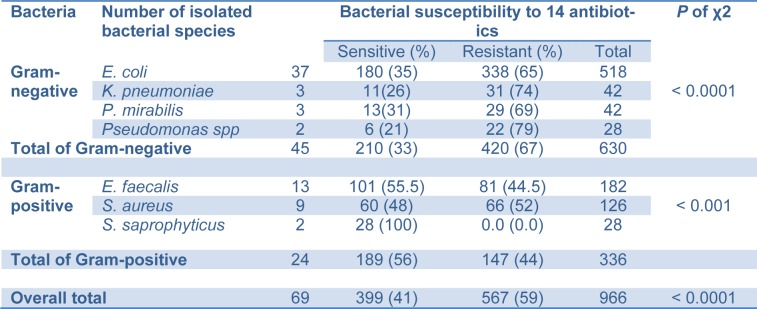
Comparison between numbers of susceptible and resistant isolates to the 14 utilized antibiotics
